# Past, present, and future predictions on the suitable habitat of the Slender racer (*Orientocoluber spinalis*) using species distribution models

**DOI:** 10.1002/ece3.9169

**Published:** 2022-07-30

**Authors:** Il‐Kook Park, Amaël Borzée, Jaejin Park, Seong‐Hun Min, Yong‐Pu Zhang, Shu‐Ran Li, Daesik Park

**Affiliations:** ^1^ Division of Science Education Kangwon National University Chuncheon Korea; ^2^ Laboratory of Animal Behavior and Conservation College of Biology and the Environment, Nanjing Forestry University Nanjing China; ^3^ College of Life and Environmental Sciences Wenzhou University Wenzhou China

**Keywords:** climate change, continental climate, East Asia, glacial period, *Orientocoluber spinalis*, species distribution model

## Abstract

Species distribution models (SDMs) across past, present, and future timelines provide insights into the current distribution of these species and their reaction to climate change. Specifically, if a species is threatened or not well‐known, the information may be critical to understand that species. In this study, we computed SDMs for *Orientocoluber spinalis*, a monotypic snake genus found in central and northeast Asia, across the past (last interglacial, last glacial maximum, and mid‐Holocene), present, and future (2070s). The goal of the study was to understand the shifts in distribution across time, and the climatic factors primarily affecting the distribution of the species. We found the suitable habitat of *O. spinalis* to be persistently located in cold‐dry winter and hot summer climatic areas where annual mean temperature, isothermality, and annual mean precipitation were important for suitable habitat conditions. Since the last glacial maximum, the suitable habitat of the species has consistently shifted northward. Despite the increase in suitable habitat, the rapid alterations in weather regimes because of climate change in the near future are likely to greatly threaten the southern populations of *O. spinalis*, especially in South Korea and China. To cope with such potential future threats, understanding the ecological requirements of the species and developing conservation plans are urgently needed.

## Graphical abstract

We predicted the habitat suitability of Orientocoluber spinalis, a monotypic snake genus found in central and northeast Asia, across past, present, and future timelines, providing insights on the current distribution and their reaction to climate change.

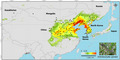

## 1 INTRODUCTION

Various anthropogenic factors are responsible for the current rapid climate change (Houghton, 1996; Teixeira & Arntzen, 2002) threatening global biodiversity (Botkin et al., 2007; Mooney et al., 2009). Over the last 8000 years, the average annual temperature in Asia rose by about 1°C, but 0.6°C of this increase happened during the last 100 years, showing an acceleration in the pattern (Ennis & Marcus, 1996; Hughes, 2000; Walther et al., 2002). As a result of climate change, species abundance change (Ehrlén & Morris, 2015; Parmesan & Yohe, 2003; Root et al., 2003; Thomas et al., 2004), distribution range shift (Bellard et al., 2012; Habibzadeh et al., 2021), and extinction rate of populations (Bestion et al., 2015; Thomas et al., 2004) are greatly impacted and might strongly fluctuate. Climate change is expected to further accelerate (Perkins et al., 2012), resulting in even more severe impacts on numerous species (Pearce et al., 1996).

Reptiles are one of the animal groups sensitive to environmental alterations because of their ectothermic characteristics and low dispersal ability (González‐Fernández et al., 2018; Huey, 1982), and they consequently face greater threats (Sinervo et al., 2010; Urban, 2015). Considering the worldwide reptile decline following the acceleration in climate change (Reading et al., 2010; Winne et al., 2007), developing conservation plans to prevent the decline of species is necessary. A step in this direction, and in particular for reptile groups with small population size, is to conduct basic ecological research such as determining species' distribution and the underlying environmental factors. In addition, predicting the species response to climate change is also essential to understand potential threats to the species and developing long‐term conservation plans.

Species distribution models (SDMs) are used to determine species distribution and can often predict habitat shifts following climate change (Broennimann et al., 2007). Reptile SDMs can also be effectively applied to identify unknown reptile populations in a particular region (Franklin et al., 2009; Guisan & Hofer, 2003; Raxworthy et al., 2003), such as done with *Uma inornata* (Barrows et al., 2010), *Lacerta lepida*, *Iberolacerta monticola*, *Hemidactylus turcicus* (Ceia‐Hasse et al., 2014), *Calodactylodes aureus* (Srinivasulu & Srinivasulu, 2016), and *Gekko japonicus* (Kim, Park, Bae, et al., 2020; Kim, Park, Fong, et al., 2020). SDMs for past timelines can also provide ecological and evolutionary information on the historical shifts of species distribution over time (Acevedo et al., 2012; Elith & Leathwick, 2009; Franklin, 2010; Teixeira & Arntzen, 2002). Specifically, past models can be used to predict historical hot spots and potential migration routes (Carnaval & Moritz, 2008; Nogués‐Bravo, 2009; Ruegg et al., 2006) and to explain phylogeographic patterns and speciation processes (Kim, Park, Fong, et al., 2020; Nogués‐Bravo, 2009; Waltari & Guralnick, 2009). In addition, research on the adaptation of species to past climate change provides a significant perspective on how species will react to climate change in the future (Pearson, 2006). Models about future distributions provide information on the likely range shift of current populations and predictions about habitat suitability (Barrows et al., 2010; Nogués‐Bravo, 2009; Sinclair et al., 2010). SDMs are thus becoming an increasingly important tool for ecology, evolution biology, conservation biology, and climate change biology (Botkin et al., 2007; Guisan et al., 2013; Mi et al., 2017; Zhang et al., 2011). Specifically, these modeling approaches across past, present, and future can be most important to understand the status of threatened, rare, and less known species (Araújo et al., 2004; Fois et al., 2018; Sinclair et al., 2010).

The Slender racer (*Orientocoluber spinalis*) is the only species within the genus *Orientocoluber*. The species was recently transferred from the genus *Hierophis* to *Orientocoluber* based on morphological, osteological, and biogeographical characteristics (Kharin, 2011; Nagy et al., 2004). This species has a wide distribution through central Asia and northeast Asia including the Republic of Korea (hereafter, South Korea), the Democratic People's Republic of Korea (North Korea), the People's Republic of China (China), Mongolia, the Russian Federation (Russia), and the Republic of Kazakhstan (Kazakhstan; Kharin & Akulenko, 2008; Munkhbayar & Munkhbaatar, 2012). Field observations of the species are very rare across all countries (Kharin & Akulenko, 2008; Kim & Han, 2009), and *O. spinalis* is listed as a threatened species in South Korea (Ministry of Environment, 2017). Up to date, the distribution and the environmental factors affecting the species habitat suitability are not known, hindering ecological studies and conservation actions.

In this study, we performed SDMs using environmental variables across the entire range of *O. spinalis* to predict past and current distributions and to identify the climatic factors key to the distribution of the species. In addition, we predict a shift in the habitat suitability of *O. spinalis* following climate change. The results of our SDMs may help understand the shifts in distribution across time, and the climatic factors primarily affecting the distribution of the species as well as to determine the origin and historical dispersal of the species.

## 2 MATERIALS AND METHODS

### 2.1 Sampling

To analyze the ecological requirements and conduct SDMs on *Orientocoluber spinalis*, we obtained presence data from field surveys, reptile researchers, research institutes (National Institute of Ecology and Korea National Park), Global Biodiversity Information Facility (GBIF; www.gbif.org/species/8198940), and iNaturalist (www.inaturalist.org). Among the data from iNaturalist and GBIF, only reliable data with photographs and GPS coordinates specifying up to the 4th decimal were used for modeling. Through the processes, we obtained 254 presence locations in total, consisting of 150 datapoints in South Korea, 1 in North Korea, 98 in China, 3 in Mongolia, 1 in Russia, and 1 in Kazakhstan (Table 1).

**TABLE 1:** Presence data of *Orientocoluber spinalis* obtained. For modeling, 94 of total of 254 data were selected and used to prevent model overfitting for biased locality data over the study areas.

Country	Province	All presence	Selection
Republic of Korea	Gyeonggi	4	1
	Gangwon	5	1
	Chungcheon	49	3
	Gyeongsang	10	4
	Jeolla	75	6
	Jeju	7	1
	Total	150	16
Democratic people's Republic of Korea	Hamgyeong	1	1
	Total	1	1
People's Republic of China	Liaoning	19	14
	Shaanxi	13	8
	Gansu	11	6
	Hebei	9	8
	Inner Mongolia	9	8
	Heilongjiang	7	4
	Shandong	7	6
	Jilin	6	5
	Shanxi	5	4
	Beijing	4	2
	Ningxia Hui	3	2
	Anhui	2	2
	Henan	2	2
	Jiangsu	1	1
	Total	98	72
Mongolia	Khovd	1	1
	Omnogovi	2	2
	Total	3	3
Russian federation	Khasanskiy	1	1
	Total	1	1
Republic of Kazakhstan	Ushtobe	1	1
	Total	1	1
Total		254	94

**FIGURE 1:**
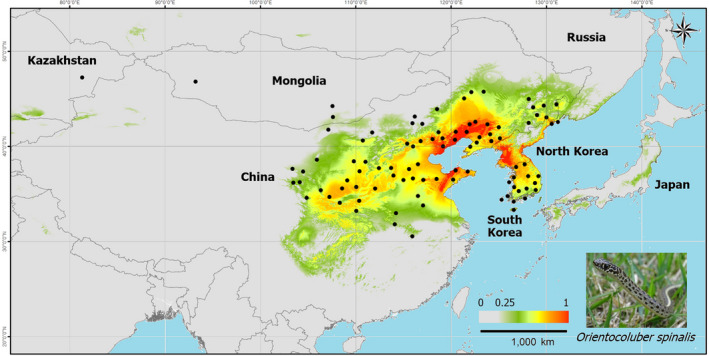
Habitat suitability of *Orientocoluber spinalis* predicted at the present time, and the location (●) of 94 *O. spinalis* presence data collected between 2006 and 2020. These datapoints were used for the species distribution models. The 0.25 (light green) is the threshold value of the suitable habitat for *O. spinalis*, and the 1 (red) indicates the most suitable habitat.

Data pre‐analysis showed that 59.1% (*n* = 150) of the presence data was congregated in South Korea, within an area accounting for only 0.36% of the total modeling areas. A bias in presence can cause overrepresentation, with the environmental variables from that particular region excessively affecting habitat suitability (Boakes et al., 2010; Kramer‐Schadt et al., 2013). To avoid overfitting of the habitat suitability of *O. spinalis* in South Korea, we spatially filtered on the data (Anderson & Raza, 2010; Radosavljevic & Anderson, 2014; Kim, Park, Bae, et al., 2020) using a 1 km, 60 km, 75 km, and 100 km radius. For each radius, we selected only one random presence datapoint among the overlapping data. We calculated the density of filtered presence datapoints and visualized their distribution with the “heatmap” tool in QGIS (QGIS.org, 2020). The distribution of the presence data was the most appropriate based on the 75 km radius when compared with the distribution of the species. Thus, for further analyses, we retained a total of 94 presence datapoints (16 in South Korea, 1 in North Korea, 72 in China, 3 in Mongolia, 1 in Russia, and 1 in Kazakhstan; Figure 1, Table 1).

### 2.2 Environmental variables

We designated East Asia (approximately 28 million km^2^; N 18.1°–54.5°, E 73.6°–149.1°) as the modeling area, including the countries where *O. spinalis* was previously recorded: South Korea, North Korea, China, Mongolia, southern Russia, and Kazakhstan. The modeling area had diverse environmental characteristics with an average annual temperature ranging from −15.6°C to 28.5°C, average annual precipitation ranging from 12 to 11,401 mm, and a maximum altitude of 8438 m above sea level. We built our model with environmental variables on a 1 km × 1 km grid (30 arc‐second resolution) consisting of the variables altitude (general bathymetric chart of the oceans; www.gebco.net) and 19 bioclimatic variables for present (1960–1990), past, and future timelines from Worldclim database (www.worldclim.com). To avoid overfitting and multicollinearity of the variables, we selected five environmental variables with correlation values lower than 0.7 among the 19 bioclimatic variables (SPSS v. 24.0, IBM Corp., 2016; Beaumont et al., 2005; Kutner et al., 2005; Zainodin & Yap, 2013; Petitpierre et al., 2017). The selected five bioclimatic variables were annual mean temperature (Bio 1), mean diurnal range (Bio 2), isothermality (Bio 3), annual precipitation (Bio 12), and precipitation seasonality (Bio 15). These variables are commonly used for reptile SDMs (Nogués‐Bravo, 2009; Pearman et al., 2008; Peterson & Nyari, 2008). In total, we used six environmental variables for the SDMs predicting the suitable habitat of *O. spinalis*, consisting of five bioclimatic variables and the altitude variable. Other ecological and abiotic variables were not consistently available for the SDMs across past, present, and future timelines and we did not include them in the modeling.

We performed the SDMs for past and future prediction based on the Community Climate System Model 4.0 (CCSM4), which is often used for amphibians and reptile SDMs (Borzée et al., 2019; González‐Fernández et al., 2018; Kim, Park, Bae, et al., 2020). For the past modeling, we used three time windows: (1) the last interglacial period about 130,000 years ago, when the average air temperature was 5°C higher and sea level was 2.2 to 3.4 m higher than present (Andersen et al., 2004; Otto‐Bliesner et al., 2006). (2) The last glacial maximum period, about 22,000 years ago, when the air temperature was about 6°C lower and the sea level was about 125 m lower than present (USGS, 2012). (3) The mid‐Holocene period, about 6000 years ago, when air temperature and the sea level were similar to the present (Gagan et al., 1998). SDMs based on these historical climatic predictions have been conducted in various animal groups, including *G. japonicus* (Kim, Park, Fong, et al., 2020), *Pelobates cultripes* (Gutiérrez‐Rodríguez et al., 2017), and *Lepus granatensis* (Acevedo et al., 2012).

As a future climate prediction model, we used the Coupled Model Intercomparison Project (CMIP5) produced by the Intergovernmental Panel on Climate Change (IPCC). This model provides four scenarios of the representative concentration pathways (RCPs), which are predicted by dividing the radiation dosage according to greenhouse gas concentrations into four stages: 2.6, 4.5, 6.0, and 8.5 W/ ^2^ (IPCC, 2014). In this study, we used RCP 4.5 and RCP 8.5 to conduct SDMs for 2070s (Archis et al., 2018; González‐Fernández et al., 2018; Kim, Park, Bae, et al., 2020). Considering applied past timeline scales, we performed the modeling only for 2070s, not including 1950s. We selected RCP 4.5 and 8.5 as they, respectively, predict that the average global air temperature will increase to 2.6°C and 4.6°C by 2100 (IPCC, 2014; Meinshausen et al., 2011).

### 2.3 Modeling methods

We performed the SDMs on *O. spinalis* using a maximum entropy modeling software (MaxEnt v. 3.4.4; Philips et al., 2020) because of its high predictive power in both extrapolation and interpolation with presence‐only data (Heikkinen et al., 2012; Phillips et al., 2006; Wisz et al., 2008). MaxEnt is one of the most efficient approaches for predicting predicting species' potential distributions (Elith et al., 2006; Elith et al., 2011; Phillips et al., 2006). We ran the models with 25% random test, 15 bootstrap replicates, 5000 iterations, one regularization multiplier, and a logistic output (Phillips, 2005; Young et al., 2011). We used the area under the curve (AUC) and the true skill statistic (TSS) to validate the model reliability using the sdm package in R (Naimi & Araujo, 2016). The AUC is based on the receiver operating curve (ROC), and it is commonly used to evaluate model performance (Allouche et al., 2006; Bradley, 1997). TSS is a simple and easy method to verify the sensitivity and specificity predictive power of SDMs (Allouche et al., 2006; Shabani et al., 2018). To determine the suitable habitat and its rate of change for *O. spinalis*, we designated a threshold using the approach called “maximizing the sum of sensitivity and specificity (max SSS)” as it is an adequate method for presence‐only data (Liu et al., 2013). In addition, we used the jackknife test and computed the response curves built into the MaxEnt software to evaluate the contributions of each environmental variable in the models (Jiménez‐Valverde, 2012). Also, to identify climate extrapolation in different periods, we used the multivariate environmental similarity surfaces (MESS) analysis incorporated into MaxEnt software (Archis et al., 2018; Elith et al., 2010). As our past and future models were built based on present climate variables (1960–1990), it is necessary to be careful with interpretations for the area outside of the present climate range (Carneiro et al., 2016). MESS analyses provide a value ranging from −100 to 100 where negative values represent regions with novel variable values, and a larger absolute negative value represents a greater difference from the present. Zero values represent variable conditions just before out of range from present Positive values represent a similarity between variables from other periods and present variables, and a positive value close to 100 is closer to present (Elith et al., 2010).

## 3 RESULTS

### 3.1 Variables for suitable habitat

**FIGURE 2:**
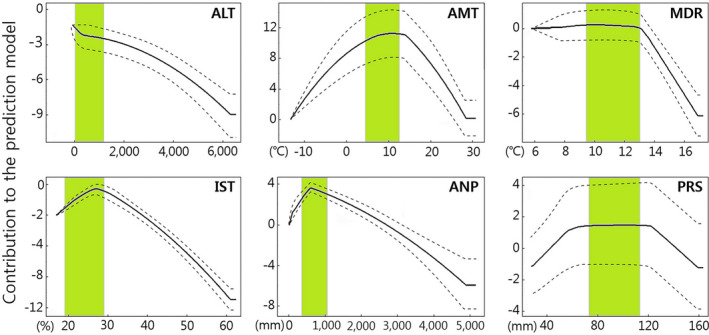
Response curves of six environmental variables in the habitat suitability modeling of *Orientocoluber spinalis*. The solid line is the mean of 15 bootstrap runs with range (dotted lines). Green bars indicate the suitable range of mean ± 1 SD values of each environmental variable, calculated from 94 presence datapoints. ALT, Altitude; AMT, annual mean temperature; ANP, annual precipitation; IST, isothermality; MDR, mean diurnal range; PRS, precipitation seasonality.

Our presence data originated from most areas of South Korea, North Hamgyeong province in North Korea, central and northeast regions of China, Khasanskiy province in Russia, Khovd and Omnogovi provinces in Mongolia, and Ushtobe province in Kazakhstan. The average values for the six environment variables selected where *O. spinalis* were observed were 613 ± 596 m of altitude, 8.6 ± 4.0°C annual mean temperature, 13.9 ± 1.8°C mean diurnal range, 26.7 ± 2.6% isothermality, 688 ± 351 mm annual precipitation, and 93 ± 20 mm precipitation seasonality. The present SDMs for *O. spinalis* were highly reliable (AUC = 0.938 ± 0.009 and TSS = 0.789 ± 0.037). The threshold value for habitat suitability calculated through max SSS was 0.537. We conducted six models for the past, present, and future, but represented only present value because all results were based on the present coordinates and variables. According to the response curves, lowlands with an annual mean temperature of around 10°C and annual precipitation of around 600 mm were critical for the suitable habitat of *O. spinalis* (Figure 2). The contribution of each variable to the model was in the order of annual mean temperature (49.8%), isothermality (16%), annual precipitation (14%), precipitation seasonality (10.4%), altitude (6.6%), and mean diurnal range (3.3%).

### 3.2 Climate similarity

**FIGURE 3:**
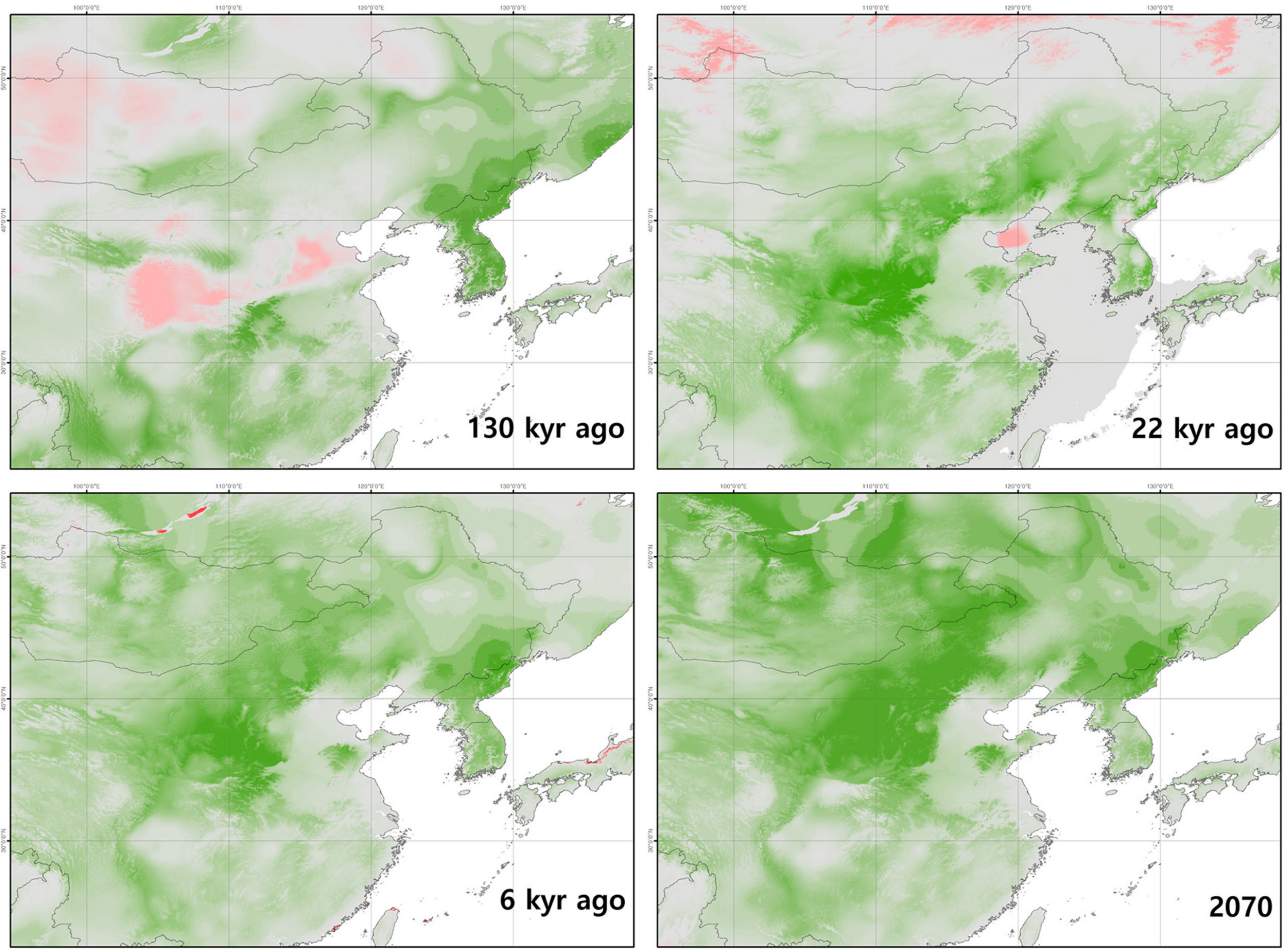
Climate similarity to the present of four eras (last interglacial period 130 kyr ago, last glacial maximum period 22 kyr ago, mid‐Holocene period 6 kyr ago, and near future 2070) performed using multivariate environmental similarity surfaces (MESS) analysis. MESS analysis was performed based on five types (annual mean temperature, mean diurnal range, isothermality, annual precipitation, and precipitation seasonality) of climate variables used to build models, and green, gray, and red indicate positive (similar climate to present), zero (just before out of range from present climate), and negative (novel climate to present) values, respectively. Darker color means higher absolute value.

The modeled climate for 6 kyr ago and 2070 did not generally deviate from the present climate range; however, several regions were dissimilar 130 and 22 kyr ago, and warrant caution when interpreting the data (Figure 3). The climate of central China and Mongolia 130 kyr ago was quite different from present, but the climate of the Korean Peninsula was very similar to present. The areas around the Yellow Sea 22 kyr ago had a similar climate to present, but the Bohai Sea area, which had become land in the Glacial age, and Russia were very different from present. The climate 6 kyr ago was generally similar to present, and we found dissimilarities only in the northern coast of Japan and Baikal Lake areas. In the 2070s projections, the northern area, especially North Korea, north‐central China, and northeast Mongolia had the highest climate similarity to present.

### 3.3 Distribution shift

According to the present model results (1960–1990), the suitable habitat of *O. spinalis* over that time period was mainly distributed in the Korean Peninsula and the east coast to the central regions of China around the Yellow Sea, at the exception of southern regions (south of the Yangtze River) in China (Figure 1). Most regions in the Korean Peninsula, except for the high‐altitude area of the Baekdudaegan Mountain Range in the northeastern area, were suitable for *O. spinalis*. We determined the presence of previously unknown suitable habitats in the mid‐southern regions of China such as Sichuan, Chongqing, Guizhou, and Hubei. In Russia, southern Khasanskiy was the only region with suitable habitat for *O. spinalis*. The distribution of suitable habitat for *O. spinalis* was sparse in both southern Mongolia and eastern Kazakhstan.

**FIGURE 4:**
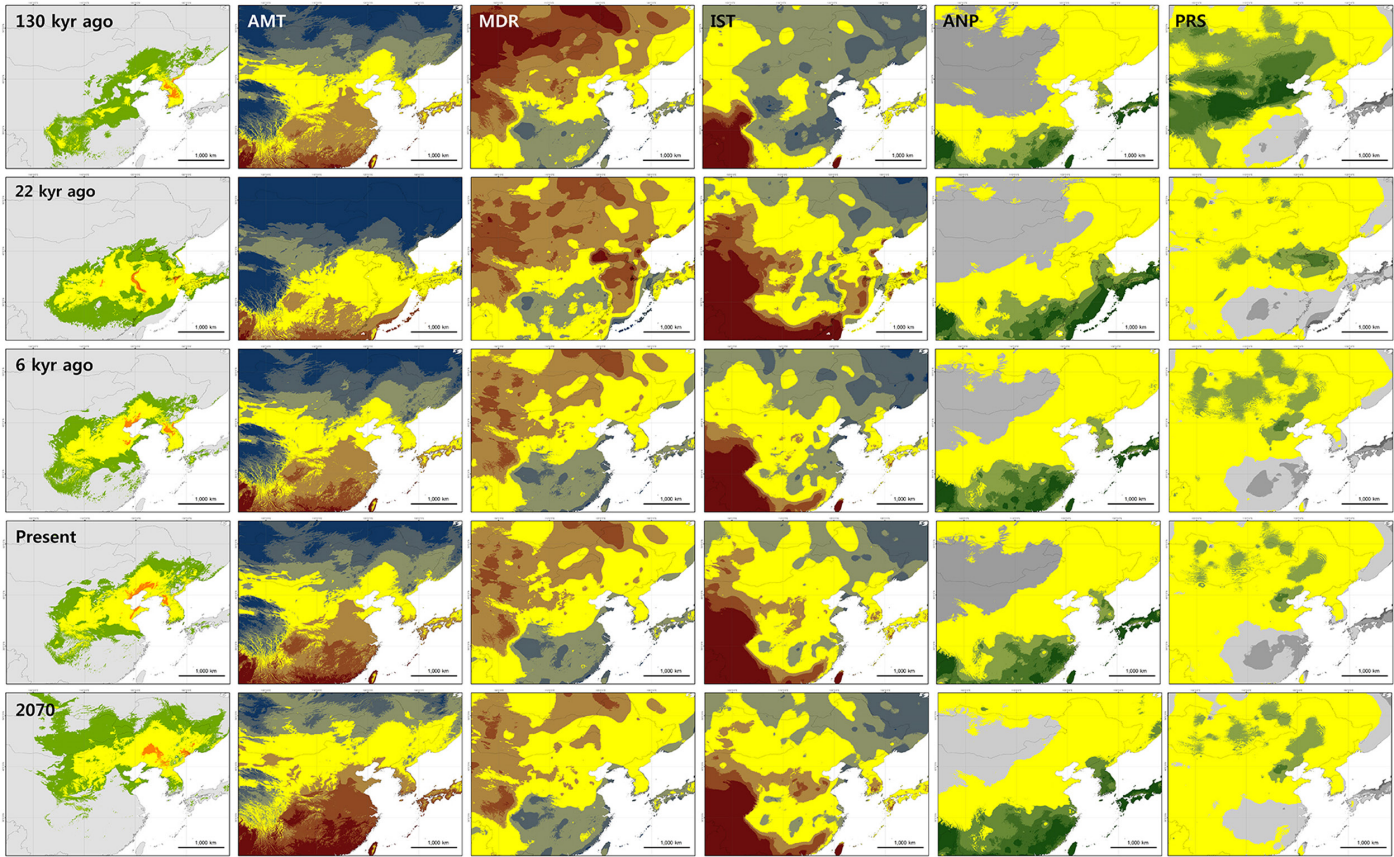
Habitat suitability of *Orientocoluber spinalis* predicted across five different timelines (last interglacial period 130 kyr ago, last glacial maximum period 22 kyr ago, mid‐Holocene period 6 kyr ago, present, and near future 2070). Suitable conditions of five climate variables were shown in parallel. (1) In suitable habitat panels (most left), different colors indicate different habitat suitability: gray: unsuitable, green: low suitable, yellow: suitable, and orange: high suitable habitat. (2) In the panels of three temperature‐related variables (annual mean temperature, AMT; mean diurnal range, MDR; isothermality, IST), dark blue: low, blue: low but weak, yellow: appropriate, red: high but available, and dark red: very high conditions for suitable habitat. (3) In the panels of two precipitation‐related variables (annual precipitation, ANP; precipitation seasonality, PRS), dark gray: low, gray: low but weak, yellow: appropriate, green: high but available, and dark green: very high conditions for the suitable habitat of *O. spinalis*.

**FIGURE 5:**
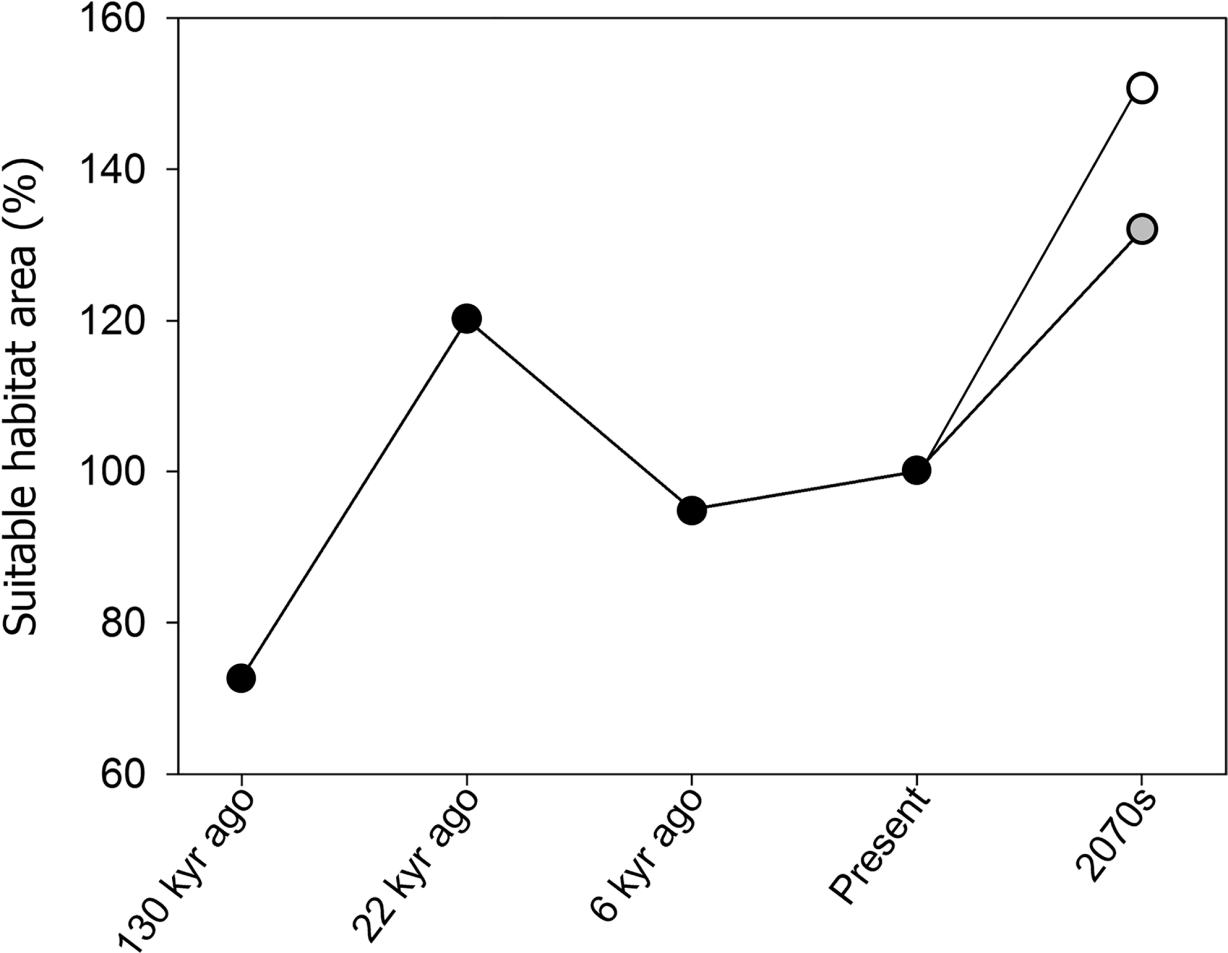
Changes in the suitable habitat areas of *Orientocoluber spinalis* predicted across past, present, and future. The values of the past and future were standardized to the present suitable habitat area. In 2070s, each gray and white circle represents RCP 4.5 and RCP 8.5 scenarios, respectively.

During the last interglacial, 130 kyr ago, 72.8% of the suitable habitat of *O. spinalis* was concentrated on the current Korean Peninsula area, and south‐central and eastern areas of current China (Table 2; Figure 4). Specifically, habitat suitability was the highest in the mid‐central areas of the Korean Peninsula. During the last glacial maximum, 22 kyr ago, the suitable habitat of *O. spinalis* had expanded to the southern regions of the Korean Peninsula and mid‐central and mid‐eastern coastal areas of China, covering an area 120.1% larger than the present range area (Table 2; Figure 5). Especially, southern regions of the Korean Peninsula and the area along the eastern coastline of China were qualified by high habitat suitability. During the mid‐Holocene, 6 kyr ago, the area with suitable habitat for *O. spinalis* covered 95.0% of the present suitable habitat for the species (Figure 5), and 84.4% of the suitable habitat overlapped with the present suitable habitats. During this time period, the most suitable habitat was located in mid‐central Korea and northern China.

**TABLE 2:** Changes in the suitable habitat of *Orientocoluber spinalis* predicted across past, present, and future. The relative and overlapped areas were standardized to the present result on suitable habitat area.

Timeline	Area (km^2^)	Relative area to the present (%)	Overlapped area to the present (%)
130 kyr ago	3,012,418	72.8	59.9
22 kyr ago	4,966,402	120.1	51.8
6 kyr ago	3,926,934	95.0	84.4
Present	4,135,616	100.0	100.0
2070 RCP 4.5	5,456,636	131.9	80.8
2070 RCP 8.5	6,229,675	150.6	69.1

Following the 2070s predictive models, and compared to the current values, the suitable habitat area for *O. spinalis* increased to 131.9% under the RCP 4.5 scenario and 150.6% under the RCP 8.5 scenario (Figure 5). In both models, the predicted suitable habitat shifted northward; reaching northeast and northern China, central and eastern Mongolia, southern Russia, and eastern Kazakhstan (Figure 4). The suitable habitat overlapped with 80.8% of the present suitable habitat following the RCP 4.5 and 69.1% following the RCP 8.5%.

## 4 DISCUSSION

Here, we show that *O. spinalis* has a wide distribution, unlike related species that occur across a relatively restricted range (Das et al., 2019; Mirza et al., 2016; Nagy et al., 2004). For example, *Hierophis* spp. mainly inhabit southern Europe, a warm Mediterranean climate (Jablonski et al., 2017; Rato et al., 2009) and *Eirenis* spp. are limited to the dry climate of the Middle East, northeastern Africa, and southern Europe (Candan et al., 2019; Mahlow et al., 2013). Our results suggest that *O. spinalis* may have a high ecological tolerance, enabling the species to have adapted and survived through glacial periods in northeast Asia.

The suitable habitat of *O. spinalis* covers the continental climate regions that are neither too cold nor too humid (Kottek et al., 2006; Peel et al., 2007), with an annual mean temperature of around 9°C and annual precipitation of around 700 mm. The continental climate with high habitat suitability for the species is also characterized by a large annual temperature variation (Kottek et al., 2006; Peel et al., 2007). Therefore, appropriate isothermality could be another critical variable for habitat suitability. In our modeling of present times, northern and southern suitable habitat boundaries were largely determined by annual mean temperature and precipitation variables. The Korean Peninsula and mid‐east coastal and mid‐inland areas of China around the Yellow Sea were the most suitable habitat for *O. spinalis*, under the influence of the monsoon regime and hot summer humid continental climate to semiarid climate area (Peel et al., 2007). In opposition, the high‐altitude mountain areas and dried mid‐west regions of China were not adequate to the species.

The differences in climates between 130 kyr ago and 22 kyr ago for some areas necessarily result in caution when interpretating the results (Figure 3). However, the novel climate in these areas will not cause major problems in interpretation as those areas are located far from the suitable habitat of *O. spinalis*, except for central China 130 kyr ago. During the last interglacial period, the most suitable habitat of *O. spinalis* was on the Korean Peninsula. During this period, northeast Asia had an average mean temperature similar to present days but had less adequate mean diurnal range, isothermality, and annual precipitation conditions in relation to the suitable habitat (Anderson & Raza, 2010; Otto‐Bliesner et al., 2006). Specifically, large deserts due to the low precipitation regime were located in northern and northwest current China (Porter, 2001; Yang & Ding, 2008), and the unsuitable conditions of isothermality and precipitation seasonality largely extended to most areas of mid‐east to eastern coastal areas of current China, resulting in the regions not matching with the ecological requirement of the species. As a result, the suitable habitat for *O. spinalis* was restricted to Sichuan, Guizhou, Hubei, Henan, Shandong, Hebei, Liaoning, and Inner Mongolia provinces in current China (Figure 4). In opposition, suitable climate conditions for *O. spinalis* were found throughout the Korean Peninsula, with an annual mean temperature of around 10.0°C and about 1100 mm of annual precipitation.

During the last glacial maximum period, the suitable habitat for *O. spinalis* greatly expanded and was consistently found from current mid‐central China to the eastern coastal areas of the Korean Peninsula. The better conditions of annual mean temperature, mean diurnal range, and isothermality for *O. spinalis* were found in the mid‐southern regions of current China and the oceanic areas of the current Yellow Sea, an emerged land at that time due to lower sea level (USGS, 2012). During this period, the basin of the current Yellow Sea and the Korean Peninsula were covered with dry steppe grasslands while current eastern China was covered with forest‐steppe vegetation (d'Alpoim Guedes et al., 2016; Kubatzki & Claussen, 1998; Ray & Adams, 2001), both of which conditions were suitable habitat for *O. spinalis*. Habitats in the northern areas of the Korean Peninsula and southern areas of current China below 25° latitude were not suitable habitats for *O. spinalis* at that period due to the low annual mean temperature and the excessive annual precipitation (Figure 4). Considering that *O. spinalis* is not currently found in Japan and considering the absence of known fossils, the species might not have been present in current Japan during this period. This absence despite suitable habitats for *O. spinalis* in current Kyushu is linked to the fact that a complete bridge between the Asian mainland and Japan is unlikely to have formed, or for a very temporary period (d'Alpoim Guedes et al., 2016; Pinxian & Xiangjun, 1994).

During the mid‐Holocene, the distribution of the suitable habitat for *O. spinalis* was similar to that of present times, with 84.4% overlap. Around 15 kyr ago, the Yellow Sea between the Korean Peninsula and current China was filled due to the increase in sea levels following an increase in air temperature (d'Alpoim Guedes et al., 2016). In current China, the suitable habitat gradually shifted northward, into Gansu, Shaanxi, Shanxi, Henan, Hebei, Shandong, Beijing, Tianjin, and Liaoning provinces. The suitable habitat in the Korean Peninsula and current China reconnected in northern Pyongan province in current North Korea and Liaoning in current China. The *O. spinalis* populations in Jeju Island and on islands on the southern and western coastal areas of the Korean Peninsula might have been established during this period. At present, *O. spinalis* is only observed in Dalnevostochny Morskoy Nature Reserve, the southern region of the Russian Far East (Kharin & Akulenko, 2008; Maslova, 2016). Our result suggests that this population might be linked to the eastern Korean Peninsula but segregated by geological and climate barriers, as recently shown for the *Bombina orientalis* in northeast China (Yu et al., 2021).

The models for 2070 have the highest climatic similarity with the present, therefore enabling the most reliable interpretation. Following our models based on future climate change scenarios, the suitable habitat for *O. spinalis* is likely to greatly expand northward in 2070s. In the RCP 4.5 and RCP 8.5 scenarios, the suitable habitat of *O. spinalis* was expected to increase by about 136.4% and 147.6%, respectively, compared to the present. The overlapping area will, however, decrease by 82.7% and 73.1%, respectively. The overall increase in air temperature in the northeastern coastal and northern inland regions of China is the main variable responsible for such changes. Climate change resulted in the migration or change in the distribution of many species (Hughes, 2003; McLeman & Smit, 2006; Pearson, 2006), and more than 68% of species are likely to see their habitat shift poleward (Karl et al., 1996; Thomas, 2010; Walther et al., 2002). Regionally, mid‐southern areas of the Korean Peninsula will lose suitable habitat for *O. spinalis* by 2070. SDMs studies on species distributed in South Korea such as *G. japonicus* (Kim, Park, Bae, et al., 2020), *Karsenia koreana* (Borzée et al., 2019), and *Onychodactylus koreanus* (Shin et al., 2021) also showed that the suitable habitat will be shrunk gradually because of climate change. Considering that the *O. spinalis* populations are mainly distributed in southern Jeolla province, South Korea (Table 1), the threats to the species in South Korea might be severe by 2070 due to shrinking of suitable habitats in the region. On the contrary, most areas in North Korea will be more suitable for *O. spinalis* by 2070, except for high elevations such as the Hamgyong and Macheonryeon Mountains. In Russia, the suitable habitat will increase by 12.8 and 8.9 times under the RCP 4.5 and 8.5 scenarios, respectively. Such an increase is also expected in Mongolia and Kazakhstan, and the species may colonize the newly available habitat.

Our results show that *O. spinalis* have overall adapted to the continental climate and their suitable habitat in response to past climate changes. In the future, we expect large range expansions and rapid northward shifts of the suitable habitat for *O. spinalis* due to the rapid contemporary climate change in comparison with past variations, potentially resulting in an increase in the threats to *O. spinalis* at low latitude in China and South Korea. Also, our results suggest that additional phylogeographic studies across China, Mongolia, and North and South Korea can further improve our understanding of the historical dispersal and distribution changes of *O. spinalis* in northeast Asia. Finally, intensive field surveys and developing population conservation plans are urgently necessary to determine and cope with future threats.

## AUTHOR CONTRIBUTIONS

**Il‐Kook Park:** Conceptualization (equal); data curation (equal); formal analysis (lead); investigation (equal); methodology (equal); validation (equal); visualization (equal); writing – original draft (equal); writing – review and editing (equal). **Amaël Borzée:** Conceptualization (supporting); methodology (equal); validation (equal); writing – review and editing (equal). **Jaejin Park:** Investigation (equal); validation (equal); visualization (equal); writing – review and editing (equal). **Seong‐Hun Min:** Investigation (equal); writing – review and editing (equal). **Yong‐Pu Zhang:** Data curation (equal); writing – review and editing (equal). **Shu‐Ran Li:** Data curation (equal). **Daesik Park:** Conceptualization (equal); funding acquisition (lead); methodology (equal); project administration (lead); supervision (lead); validation (equal); writing – original draft (equal); writing – review and editing (equal).

## CONFLICT OF INTEREST

None declared.

## Data Availability

The input data and output data in this study are accessible at Dryad Digital Repository. DOI: Dryad https://doi.org/10.5061/dryad.b5mkkwhfw.
